# A Prediction Model for Recognizing Strangulated Small Bowel Obstruction

**DOI:** 10.1155/2018/7164648

**Published:** 2018-03-26

**Authors:** Xiaming Huang, Guan Fang, Jie Lin, Keyu Xu, Hongqi Shi, Lei Zhuang

**Affiliations:** ^1^Department of General Surgery, The First affiliated Hospital of Wenzhou Medical University, Wenzhou, China; ^2^Department of Neurology, The First Affiliated Hospital of Wenzhou Medical University, Wenzhou, China

## Abstract

**Introduction:**

Early and accurate diagnosis of strangulated small bowel obstruction (SSBO) is difficult. This study aimed to devise a prediction model for predicting the risk of SSBO.

**Materials and Methods:**

A database of 417 patients who had clinical symptoms of intestinal obstruction confirmed by computed tomography (CT) were evaluated for inclusion in this study. Symptoms and laboratory and radiologic findings of these patients were collected after admission. These clinical factors were analyzed using logistic regression. A logistic regression model was applied to identify determinant variables and construct a clinical score that would predict SSBO.

**Results:**

Seventy-six patients were confirmed to have SSBO, 169 patients required surgery but had no evidence of intestinal ischemia, and 172 patients were successfully managed conservatively. In multivariate logistic regression analysis, body temperature ≥ 38.0°C, positive peritoneal irritation sign, white blood cell (WBC) count > 10.0 × 10^9/L, thick-walled small bowel ≥3 mm, and ascites were significantly associated with SSBO. A new prediction model with total scores ranging from 0 to 481 was developed with these five variables. The area under the curve (AUC) of the new prediction model was 0.935.

**Conclusions:**

Our prediction model is a good predictive model to evaluate the severity of SBO.

## 1. Introduction

Strangulated small bowel obstruction (SSBO) may lead to intestinal perforation, ischemia, and necrosis mainly due to compromised blood flow [1, 2]. It is reported that SBO accounts for 12% to 16% of surgical admissions, and no less than 300,000 surgical operations are performed in the United States every year [3]. A 35-year institutional experience revealed 42% of small bowel obstructions (SBO) to be due to strangulation. Meanwhile, nonviable strangulation accounts for 16% of SBOs, which have a fourfold increase in the risk of death compared to viable strangulation [4]. Another study reported that patients with strangulated obstruction have 2 to 10 times higher rates of death than those with nonstrangulated obstruction [5, 6]. Thus, in order to prevent strangulation and potential bowel necrosis leading to higher morbidity and mortality rate, prompt differentiation of the characteristics of SBO is needed [6, 7]. Strangulated obstruction may require immediate surgical intervention [5].

In-hospital mortality in patients who underwent emergency gastrointestinal (GI) surgery was associated with cancer-related peritonitis, preoperative anemia, and preoperative hypoalbuminemia [8]. Older patients older than 90 years who underwent emergency surgery had a higher mortality rate than younger patients [9]. Furthermore, some studies showed that a large number of non-SSBO cases could be successfully managed with conservative treatment [7, 10, 11]. Therefore, to avoid the risk of emergency surgery, patients without SSBO should be identified and managed conservatively.

Clinical parameters, including medical history and physical examination, laboratory test, and imaging findings can provide a better evaluation of the risk of underlying bowel strangulation and help to establish an appropriate plan of SBO treatment [12, 13].

In this study, we aimed to devise a model for predicting the risk of SSBO.

## 2. Methods

### 2.1. Study Population and Data Collection

The study protocol was approved by the Institutional Review Board of the First Affiliated Hospital of Wenzhou Medical University, and it conformed to the concepts of the declaration of Helsinki and its amendments. Between January 2007 and December 2015, 417 patients from The First Affiliated Hospital of Wenzhou Medical University with clinical symptoms of intestinal obstruction confirmed by CT were evaluated for inclusion in the study. Patients with large bowel obstruction, inguinal hernia, early postoperative SBO (occurring less than 30 days after abdominal operation), malignancy, and patients with a known history of ascites were excluded from this study.

Collected clinical parameters during admission included age, sex, past history of abdominal surgery, duration of hospital stay, relevant admission symptoms (vomiting), body temperature, and heart rates. Collected results of laboratory tests included white blood cell (WBC) count. We also recorded physical signs detected by the surgeon (peritoneal irritation signs as tenderness, rebound, and guarding). Abdominal computed tomography (CT) scans were also recorded. The patients with symptoms and signs of obstruction underwent noncontrast CT scan when they arrived in our hospital. All abdominal CT scans were reviewed by radiologists, and all imaging reports were completed before the operation. The CT parameters included small bowel dilatation (>4 mm), thick-walled small bowel (>3 mm), ascites, small bowel air fluid level, and volvulus.

There were three clinical outcome categories: patients with SBO and successful conservative treatment until discharge, patients who underwent operation but had no evidence of intestinal ischemia, and patients who underwent urgent laparotomy with evidence of intestinal ischemia requiring small bowel resection. The diagnosis of ischemic small bowel was confirmed by pathological examination of the surgical specimen.

In patients who had no evidence of intestinal ischemia, the indication to operate was ambiguous, and these data were not used in the analysis of predictive factors.

### 2.2. Statistical Analysis

Statistical analyses were performed using Statistical Package for Social Sciences (SPSS) version 17.00 (Chicago, IL, USA). Continuous variables were divided into clinically meaningful categories and compared between the three patient groups using chi-square and Fisher's exact tests.

The clinical variables were assessed by univariate logistic regression models and were summarized by regression coefficient, an odds ratio (OR), and 95% confidence interval (CI). All variables with univariable *P* < 0.05 were considered for the multivariate model. All variables with an adjusted *P* < 0.05 were retained in the final model. These variables were finally determined with a backward stepwise variable selection. A clinical score was constructed based on the final logistic regression model, and this predictive score system was determined by logarithmically transforming it for each selected variable and multiplying by 100 (*Y* = 100 × log *X*, where *Y* is the score and *X* is the odds ratio).

To assess the discriminative ability of this model, a receiver operating characteristic (ROC) curve was obtained and the area under the curve (AUC) was calculated. We used AUC to evaluate the predictive ability of this multivariable model. The AUC ranged from 0.5 to 1.0, with values of 0.5 indicating no predictive ability and 1.0 indicating a perfect predictive ability. *P* values of less than 0.05 were considered statistically significant. The sensitivity and specificity of each threshold of the score were examined.

## 3. Results

### 3.1. Etiology and Characteristics of Patient

The etiology of patients is described in Table 1. Volvulus is the primary etiology in patients with SSBO, and the adhesive disease is the second. Adhesive disease is the primary etiology in patients with non-SSBO.

A total of 417 patients with SBO were included in the study; 76 (18.1%) were confirmed to have SSBO, 169 (40.8%) patients required operation but had no evidence of intestinal ischemia, and 172 (41.1%) were successfully managed conservatively. Men comprised 277 and women comprised 140 patients. Average patient age was 57.4 (range 16–88) years. Two patients with SSBO died of entire small bowel necrosis.

The average duration from admission to operation in patients with SSBO was 57.41 hours. The duration of hospital stay was 1–6, 7–13, and 14–103 days. There were statistically significant differences between the three groups; patients who underwent surgery stayed in the hospital for a longer duration than those treated conservatively (*P* < 0.001). Clinical and laboratory parameters, physical examination, and CT findings of patients with SSBO are summarized in Table 2.

### 3.2. Comparison between Conservative and SSBO Groups

Patients who underwent either conservative treatment or surgery for strangulated SBO were compared after exclusion of the group that underwent surgery but had no evidence of intestinal ischemia.

#### 3.2.1. Univariate Analysis

Univariate associations of clinical and laboratory parameters, physical examination, and CT findings of patients with SSBO are summarized in Table 3. On univariate analysis, SSBO was significantly associated with body temperature ≥ 38.0°C, peritoneal irritation sign, WBC (×10^9/L) > 10.0, and CT: thick-walled small bowel ≥3 mm, small bowel air fluid level, ascites, and volvulus.

In contrast, body temperature ≥ 38.0°C was associated with a 3.8-fold increased risk of strangulated obstruction. Positive peritoneal irritation sign was associated with a 34-fold increased risk of strangulated obstruction. WBC count > 10.0 × 10^9/L was associated with a 4.5-fold increased risk of strangulated obstruction. The presence of ascites on CT was associated with more than 24-fold increased risk of strangulated obstruction. The presence of thick-walled small bowel ≥3 mm on CT was associated with more than 16-fold increased risk of strangulated obstruction. The presence of volvulus on CT was associated with more than 2.7-fold increased risk of strangulated obstruction. However, the presence of small bowel air fluid level on CT was associated with more than 0.4-fold increased risk of strangulated obstruction. The results are listed in Table 3.

#### 3.2.2. Multivariate Analysis

**Figure 1:**
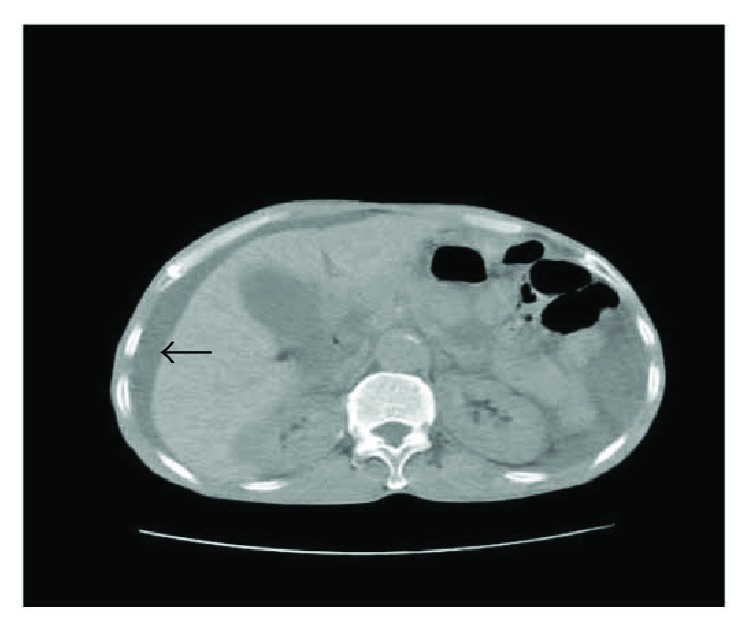
Computed tomography scan of the abdomen showing a large quantity of ascites (black arrow) around the liver.

Multivariate logistic regression analysis showed that the findings significantly associated with SSBO were body temperature ≥ 38.0°C, peritoneal irritation sign, WBC (×10^9/L) > 10.0, and CT: thick-walled small bowel ≥ 3 mm and ascites (Figure 1). The results are shown in Table 4. Patients with WBC count > 10.0 × 10^9 were 4.5 times more likely to have SSBO. Body temperature ≥ 38.0°C was associated with a greater than 6-fold increased risk for SSBO. Positive peritoneal irritation sign was associated with a greater than 13-fold increased risk for SSBO. Thick-walled small bowel ≥3 mm and ascites were associated with a 11.0- and 16.8-fold increased risk, respectively.

### 3.3. A New Prediction Model for SSBO

**Figure 2:**
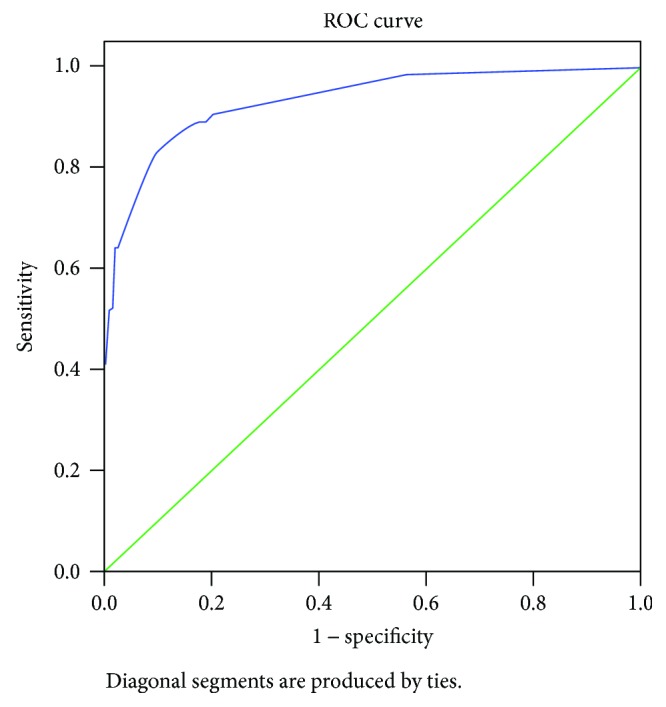
Receiver operating characteristic (ROC) curve for the prediction model. The area under the curve was 0.935 (95% CI, 0.900–0.969).

This prediction model was developed by logarithmically transforming each selected variable and multiplying by 100 (*Y* = 100 × log *X*, where *Y* is … and *X* = odds ratio) as shown in Table 4. The estimated rates of SSBO were calculated for the total scores ranging from 0 to ≥299 as shown in Table 5. A score ≥ 299 had 40.8% sensitivity and 100.0% specificity, a score ranging from 298 to 226 had 64.5% sensitivity and 98.3% specificity, a score ranging from 225 to 143 had 85.5% sensitivity and 88.4% specificity, a score ranging from 142 to 65 had 98.7% sensitivity and 43.0% specificity, and a score ≥ 0 had 100% sensitivity and 0% specificity. The optimal cutoff point with the highest sum of sensitivity and specificity was for the score of 132.5. The ROC curves of the logistic regression model from Table 4 and the model are shown in Figure 2. The AUC in multiple logistic regression was 0.936 (95% CI: 0.901–0.970). The AUC for the prediction model was 0.935 (95% CI: 0.900–0.969). Though the risk equation was transformed, the loss of discriminative ability was minimal.

## 4. Discussion

Strangulated obstruction is a life-threatening complication of SBO. Prompt diagnosis of SSBO and surgical intervention are important to avoid serious complications, such as perforation, sepsis, and death [6]. However, patients with poor general condition cannot tolerate emergency surgery. Therefore, in order to select an appropriate treatment plan, our study sought to devise a score that would help clinicians distinguish between simple and strangulated SBO.

A number of previous studies have evaluated the accurate and early diagnosis of strangulated SBO, but early detection remains difficult; thus, the identification of more reliable diagnostic tools is required urgently.

Animal experiments showed that CRP levels were associated with the severity of bacterial translocation in acute intestinal obstruction but did not distinguish between simple SBO and SSBO [5].

Another study showed that the D-dimer level was neither sensitive nor specific in predicting SSBO [14]. Furthermore, a series study of urinary intestinal fatty acid-binding protein (I-FABP) for predicting strangulated mechanical small bowel obstruction showed that it was a reliable marker for SSBO, but the sample size was small. Therefore, further studies evaluating the predictive power of I-FABP are needed [15, 16]. Up until now, we have no reliable laboratory marker for predicting SSBO.

CT scans for diagnosis of SSBO have been studied for many years. High sensitivity and specificity of CT scans in the diagnosis of strangulated small bowel obstruction were presented in several studies [13, 17–19]. One study reported some inherent limitations of CT scans to diagnose SSBO when conducted solely. It was also proposed that combining clinical and CT criteria could get over a lot of the CT's inherent limitations [20].

So far, a number of studies have been conducted to find out a predictive factor that can help in the appropriate management of SBO, but these studies focus on a part of the clinical scenario.

On the other hand, our study tried to identify the relevant clinical features, including laboratory parameters, physical examination, and CT scans. We found that body temperature ≥ 38.0°C, positive peritoneal irritation sign, WBC > 10.0 × 10^9/L, thick-walled small bowel ≥ 3 mm, and ascites were independent predictors of strangulated SBO. Therefore, we incorporated these five variables into a score that can be used as a new model for predicting SSBO.

Patients with score ≥ 299 had a 100 percent risk for SSBO. A score of at least 132.5 predicted SSBO with a sensitivity of 85.5% and specificity of 88.4%, with an AUC of 0.935. These results indicate that this model can help in establishing an appropriate plan for SBO treatment.

Zielinski and colleagues developed a multivariate prediction model for patients with SBO who need emergency operation [13]. However, they could not develop a multivariable model for these patients with strangulated SBO because there were too few patients with SSBO to support more than one feature in a model. Schwenter and colleagues also developed a clinic-radiological score for predicting the risk of strangulated small bowel obstruction [21]. The AUC of the clinic-radiological score in Schwenter's study was 0·87 (95% CI: 0·79 to 0·95). Our score had a higher AUC, and we also had a larger sample size.

This prediction model is very valuable at score ≥ 299, as 100% of patients required emergency surgery on at this score. In scores ranging from 298 to 226, emergency surgery was required in a large number of patients. In scores ranging from 225 to 143, half of the patients required emergency surgery. Patients with a score ≤ 142 should be dealt with carefully. In addition, patients with a score of 0 should be prioritized to conservative treatment.

In order to improve the diagnosis of SSBO, this score can also be combined with other laboratory makers, such as C-reactive protein, and I-FABP, if the condition permits. However, patients with this condition require continued surveillance and regular reassessment, and individual clinical judgment can play a decisive role.

The model could quantitatively evaluate the severity of SBO and might help us apply the appropriate approach when patients with SBO are admitted to the hospital.

There are several limitations in our work: Firstly, this is a retrospective study and our data are based on the medical extraction in our hospital. Hence, selection bias could not be completely avoided. Though the presence of volvulus on CT has statistical significance in univariate analysis, it was not available in this prediction model. Relationship between the presence of volvulus on CT and SSBO needs further research. Secondly, due to the single-center study, the model requires further validation. Further large-scale and well-designed studies are needed.

## 5. Conclusion

Body temperature ≥ 38.0°C, positive peritoneal irritation sign, WBC count > 10.0 × 10^9/L, thick-walled small bowel ≥3 mm, and ascites were the main risk factors for SSBO. Our prediction model is a good predictive model and can help in evaluating the severity of SBO and monitoring the evolution of a patient's condition after admission, allowing for the appropriate management of SBO. This new model requires further intensive studies for its validation.

## Figures and Tables

**Table 1:** Etiology of patients who underwent operation.

Parameter	Surgery, non-SSBO (*n* = 169)	Surgery, SSBO (*n* = 76)
Adhesive disease	103 (60.9%)	23 (30.3%)
Mesenteric arterial embolism	0	7 (9.2%)
Benign tumor	9 (5.3%)	1 (1.3%)
Stricture/stenosis	4 (2.4%)	0
Internal hernia	4 (2.4%)	9 (11.9%)
Volvulus	30 (17.8%)	34 (44.7%)
Intussusception	6 (3.6%)	2 (2.6%)
Intestinal bezoar	10 (5.9%)	0
Abdominal cocoon	1 (0.6%)	0
Foreign bodies	2 (1.1%)	0

**Table 2:** Comparison of clinical and laboratory parameters, physical examination, and CT findings of patients who received conservative treatment, surgery in patients without SSBO, and those with SSBO.

	Conservative (*n* = 172)	Surgery, non-SSBO (*n* = 169)	Surgery, SSBO (*n* = 76)	*P*
Sex				0.780
Males	116 (67)	109 (64)	52 (68)	
Females	56 (43)	60 (36)	24 (32)	
Age (years)				0.556
19–59	79 (46)	94 (56)	34 (45)	
60–69	39 (23)	36 (21)	18 (24)	
70–79	38 (23)	29 (17)	17 (22)	
80–89	16 (8)	10 (6)	7 (9)	
Duration of hospital stay (day)				<0.001
1–6	64	9	2	
7–13	89	58	38	
14–103	19	102	36	
Prior abdominal procedures				0.178
Yes	112 (65)	120 (71)	57 (75)	
No	60 (35)	49 (29)	19 (25)	
Vomiting				0.201
Yes	113 (66)	111 (66)	58 (76)	
No	59 (34)	58 (34)	18 (24)	
Temperature				<0.001
≥ 38.0°C	8 (5)	1 (1)	12 (16)	
< 38.0°C	164 (95)	168 (99)	64 (84)	
Heart rate (bpm)				<0.001
≥ 100	26 (15)	20 (12)	16 (21)	
< 100	146 (85)	149 (88)	60 (79)	
Peritoneal irritation sign				<0.001
Yes	4 (2)	20 (12)	34 (45)	
No	168 (98)	149 (88)	42 (55)	
WBC (×10^9/L)				<0.001
> 10.0	81 (47)	69 (41)	61 (80)	
< 10.0	91 (53)	100 (59)	15 (20)	
*CT parameter*				
Small bowel dilatation				0.053
≥ 4 mm	40 (23)	27 (16)	22 (29)	
< 4 mm	132 (77)	142 (84)	54 (71)	
Thick-walled small bowel				<0.001
≥ 3 mm	7 (4)	47 (28)	31 (41)	
< 3 mm	165 (96)	122 (72)	45 (59)	
Ascites				<0.001
Yes	20 (12)	26 (13)	58 (76)	
No	152 (88)	143 (87)	18 (24)	
Small bowel air fluid level				<0.001
Yes	143 (83)	112 (66)	49 (64)	
No	29 (17)	57 (34)	27 (36)	
Volvulus				0.02
Yes	19 (11)	29 (17)	19 (25)	
No	153 (89)	140 (83)	57 (75)	

Values in parentheses are percentages. CT: computed tomography; SSBO: strangulated small bowel obstruction.

**Table 3:** Univariate analysis for comparison of 172 patients with small bowel obstruction who underwent conservative treatment with 76 who had surgery for SSBO.

Variables	Regression coefficient	Odds ratio (95% CI)	*P* value
Sex, females (versus males)	−0.045	0.956 (0.536–1.706)	0.879
Prior abdominal procedures	0.525	1.691 (0.923–3.098)	0.089
Vomiting	0.571	1.771 (0.958–3.273)	0.068
Temperature ≥ 38.0°C	1.346	3.844 (1.501–9.840)	0.005
Heart rate (bpm) ≥ 100	0.404	1.497 (0.750–2.990)	0.252
Peritoneal irritation sign	3.526	34.000 (11.434–101.106)	<0.001
WBC (×10^9/L) > 10.0	1.519	4.569 (2.411–8.658)	<0.001
CT: small bowel dilatation ≥ 4 mm	0.359	1.432 (0.783–2.619)	0.244
CT: thick-walled small bowel ≥ 3 mm	2.787	16.238 (6.709–39.303)	<0.001
CT: small bowel air fluid level	−1.000	0.368 (0.199–0.682)	0.01
CT: ascites	3.198	24.489 (12.100–49.561)	<0.001
CT: volvulus	0.987	2.684 (1.326–5.432)	0.006

CI: confidence interval; WBC: white blood cell; CT: computed tomography.

**Table 4:** Multivariate associations in patients with strangulated small bowel obstruction (SSBO) and development of the new prediction model for SSBO.

Variables	Regression coefficient	Odds ratio (95% CI)	*P* value	Point(s)
Temperature ≥ 38.0°C	1.787	5.971 (1.346–26.486)	0.019	78
Peritoneal irritation sign	2.568	13.039 (3.272–51.965)	<0.001	112
WBC (×10^9/L) > 10.0	1.500	4.483 (1.660–12.107)	0.003	65
CT: ascites	2.819	16.768 (6.682–42.081)	<0.001	122
CT: thick-walled small bowel ≥ 3 mm	2.400	11.021 (3.661–33.180)	<0.001	104

CI: confidence interval; WBC: white blood cell; CT: computed tomography.

**Table 5:** Classification of patients with strangulated small bowel obstruction (SBBO) according to the score.

Score	Number of patients	Patients of SSBO (%)	Sensitivity (%): positive if ≥ score	Specificity (%): negative if ≥ score
≥299	31	100	40.8	100.0
298–226	21	85.7	64.5	98.3
225–143	33	48.5	85.5	88.4
142–65	88	11.4	98.7	43.0
0	75	1.3	100.0	0.0
